# Ethnobotanical Study of Medicinal Plants Used in Central Macedonia, Greece

**DOI:** 10.1155/2019/4513792

**Published:** 2019-04-01

**Authors:** Efthymia Eleni Tsioutsiou, Paolo Giordani, Effie Hanlidou, Marco Biagi, Vincenzo De Feo, Laura Cornara

**Affiliations:** ^1^Department of Pharmacognosy and Natural Products Chemistry, Faculty of Pharmacy, National and Kapodistrian University of Athens, Athens, Greece; ^2^Department of Pharmacy, University of Genoa, Viale Cembrano, 4, 16148 Genoa, Italy; ^3^Laboratory of Systematic Botany and Phytogeography, School of Biology, Aristotle University of Thessaloniki, 54124 Thessaloniki, Greece; ^4^Department of Physical Sciences, Earth and Environment, University of Siena, Via Laterina, 8, 53100 Siena, Italy; ^5^Department of Pharmacy, University of Salerno, Via Giovanni Paolo II, 132, 84084 Fisciano (Selerno), Italy; ^6^Department of Earth, Environment and Life Sciences, University of Genoa, Corso Europa 26, 16132 Genoa, Italy

## Abstract

This work provides the ethnobotanical data concerning the traditional use of medicinal plants in Macedonia region (Northern Greece), which has, up to now, been poorly investigated. The aim of the present study was to collect, analyze, and evaluate information on the use of medicinal plants among different population groups living in Central Macedonia. The study was carried out in the area of two small cities, Edessa and Naoussa, and nearby villages. The ethnobotanical data were gathered through extensive and semistructured interviews. The informants belonged to different population groups living in the study areas and were involved, at least partially, in agriculture. Together with detailed reports on each species, data were also summarized by some indices, such as Fidelity Level (FL) and Informant Consensus Factor (F_ic_). A group of 96 informants was interviewed and 87 plant taxa with medicinal uses were cited. Medicinal plants are used to treat a wide range of diseases, in particular ailments of the respiratory tract and skin disorders. The importance of the traditional use of plants to cure and prevent common and some uncommon diseases had been highlighted. About 55% of medicinal plants mentioned by the informants had been previously reported to be sold in Thessaloniki herbal market as traditional remedies. Medicinal uses of some endemic taxa had been reported, e.g.,* Satureja montana *subsp.* macedonica, *a member of the* S. montana* group restricted to Northern Central Greece,* Origanum dictamnus*, an endemic species of Crete, and six Balkan endemics, i.e.,* Achillea holosericea, Digitalis lanata, Helleborus odorus *subsp*. cyclophyllus, Sideritis scardica, Thymus sibthorpii, *and* Verbascum longifolium.* Several differences in Traditional Ethnobotanical Knowledge (TEK) were observed in relation to social and cultural components of the population. Only 7 species (*Crataegus monogyna*,* Hypericum perforatum*,* Matricaria chamomilla*,* Rosa canina*,* Sambucus nigra*,* Sideritis scardica,* and* Tilia platyphyllos*) were commonly reported by all population groups, whereas 30 out of 87 taxa (34%) were exclusively mentioned by a single group. All groups are incorporated in the local society and do not identify themselves as members of different ethnic groups, although they try to preserve their distinctiveness by keeping their traditions and dialects. Nevertheless, our data show that the knowledge regarding the medicinal plant use was rarely accompanied by preservation of linguistic diversity concerning the plant names. This work contributes to improve the knowledge on the traditional use of plants in the folk medicine of a region like Central Macedonia where different population groups live together, partially maintaining their traditions. A part of data of this paper has been presented as posted at 112° Congress of Italian Botanical Society (IPSC), Parma 20-23 September 2017.

## 1. Introduction

The rural regions of southeastern Europe represent a unique social and environmental context for ethnobotanical studies, owing to the occurrence of a large mountainous area that is recognized as a hotspot for both biodiversity and cultural/ethnic/religious diversities. Medicinal plants have represented, for thousands of years, the only remedy for various diseases. Phytotherapy still maintains an important role in the treatment of many diseases in Greece. Despite this fact, only a few studies have explored the use of plants in Greek folk medicine while, on the contrary, in the neighboring region of the Balkan Peninsula very intensive and highly effective ethnobotanical studies were carried out in the last decade ([1–5] and references therein). Ethnobotanical studies on traditional uses of plants and their products in Greece are relatively scarce. An old study concerning the knowledge of medicinal plants of Greece (Lawrendiadis, 1961) reported some data about most important plants used in folk medicine throughout Greece. More recent works have been mainly focused on the regions of Zagori [6, 7], Thessaloniki [8–10], Crete [11], Mt. Pelion [12], and more recently on the Greek Islands of North Aegean Region [13]. In contrast, the regions of Edessa and Naoussa (Central Macedonia), where this study was carried out, remain poorly explored from an ethnobotanical point of view, despite their high floristic and vegetation diversity.

Our study area, Central Macedonia, lies in the core of the ancient Macedonian state (500-168 B.C.) and is the homeland of Alexander the Great. It was conquered by the Romans (168-284 B.C.), then was a part of the Byzantine Empire, and subsequently became a part of Ottoman Empire (from 1430), and, finally, it was incorporated into the Greek state in 1917 [14]. Centuries of foreign dominance and migrations had shaped the area into a cultural and linguistic mosaic. Until the beginning of 20th century, Greeks, Slavs, Bulgarians, Turks, and Vlachs were living together. The current population composition was formed after the Neuilly Treaty (1919) and mainly after the Greek-Turkish War in Asia Minor (1919-1922) and the Lausanne Treaty (1923), when much of the Slav-speaking and all the Muslim populations left, and Greek populations from Asia Minor and Pontus moved in and settled [15]. Nowadays, the population consists mainly of five groups: (a) the Dopioi, i.e., the local people that remained after the migrations of the first quarter of 20^th^ century. Many of them (Slavophones or Slavo-Macedonians) speak a local Slavic-based dialect while others are Greek-speaking; (b) the Pontians, which come from the Greek population lived in the shores of the Black Sea since antiquity. Under the subsequent Ottoman rule, they survived relatively intact, preserving their customs and dialect (Pontian Greek), which is related to ancient Greek, until they were forced to leave their homeland (Day et al., 2002); (c) the Mikrasiates or prosfyges (meaning refugees), descendants of the Greeks of Asia Minor (Mikra Asia in Greek); (d) the Vlachs or Aromanians, people mostly living in montane region and occupied in animal husbandry, who speak a Latin-based language, having a long history of settlement in the study area. According to some historians, they are Latinized indigenous populations (Greeks, Illyrians, Thracians or Dardanias), due to the historical presence of the Roman military in the territory [16]. Many Romanian historians claim that the Aromanians were part of a Daco-Romanian migration from the north of the Danube [17, 18]; (e) others, who recently moved to the region from various parts of Greece.

One of the most prominent differences of above populations groups is in their dialects, which are still used, particularly by the older people, as a second language after contemporary Greek. All groups are incorporated in the local society and do not identify themselves as members of different ethnic groups (REF). However, they define themselves based on their origin and try to preserve their distinctiveness by keeping, in a large degree, their customs, music, dances, dialects, and cuisine [19–23], (Winnfrith, 2001). Taking into account the lack of any published ethnobotanical information as well as the great cultural diversity of Central Macedonia, our aim is to survey medicinal plants and their uses and to find if significant differences concerning the use of medicinal plants still persist among the groups of inhabitants of the area.

## 2. Materials and Methods

### 2.1. Study Area

**Figure 1:**
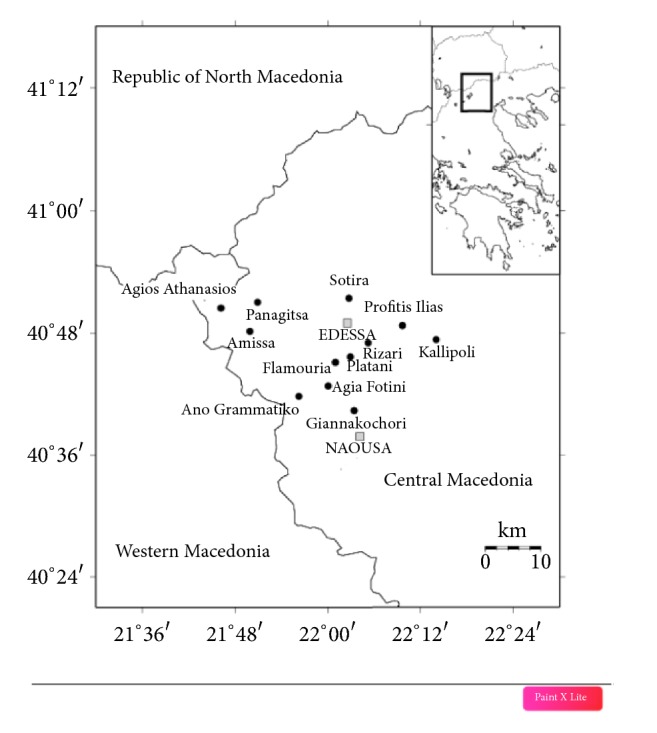
Map of Edessa, Naoussa, and the nearby villages in Central Macedonia (Greece).

Our survey was conducted in two cities, Edessa and Naousa, each with a population of c. 18.000 inhabitants and their nearby rural small villages (Figure 1). The inhabitants are mostly occupied in agriculture and stock raising. The area is located at the foothills of Mt Vermio and Mt Voras. The two mountains, which are part of the Natura 2000 Network (GR1210001 and GR1240008, respectively), are characterized by a rich and diverse flora (> 1000 taxa were recorded on Vermio, and > 1500 on Voras), including several Greek and Balkan endemic species [24, 25].

### 2.2. Methods

The fieldwork was conducted during spring and summer in 2016 and 2017. The ethnobotanical data were gathered through extensive interviews, aimed to create open informal and semistructured interviews. Snowball sampling techniques were used to recruit 96 informants (37 men, 59 women). In snowball sampling, the first contact with the community is selected as a well-known expert; in a subsequent phase, the expert indicates another expert, and so on, until all the specialists in the community are covered [26]. The informants were selected proportionally to the occurrence of five groups within the local population: (a) Dopioi (28 persons), DO (b) Pontians (22 persons), PO (c) Mikrasiates (17 persons), MI (d) Vlachs (10 persons), VL (e) others (19 persons), OTH.

In particular, in the Naoussa municipality a total of 24 informants were selected (8 OTH, 3 MI, 9 PO; 2 DO and 4 VL), 20 coming from the town and 4 from the villages. In the Edessa municipality, there were a total of 72 (11 OTH; 14 MI; 13 PO; 26 DO and 6 VL), 15 of which come from the town and 57 from 16 villages. The subdivision of the inhabitants of the area in various groups was mainly based on the different dialects that are still spoken in the two municipalities. In addition, it is worth mentioning that the various groups are diversified from each other due to different cultural characteristics such as traditional costumes, music, and folk dances. Starting from Seventies the modern way of living has prevailed. However, at the same time, there was a turning towards folklore in every county town of Greece, including Central Macedonia. Cultural societies are formed with folklore dance groups and a recovery of the traditional music is still in progress [27]. Also in the study area several different folk traditions still persist among the group population, as shown by the presence of many Folklore Museums spreading in Central Macedonia (e.g., Vlach Folklore Museum, Folklore Museum of Edessa, History and Folklore Museum of Naoussa).

**Figure 2:**
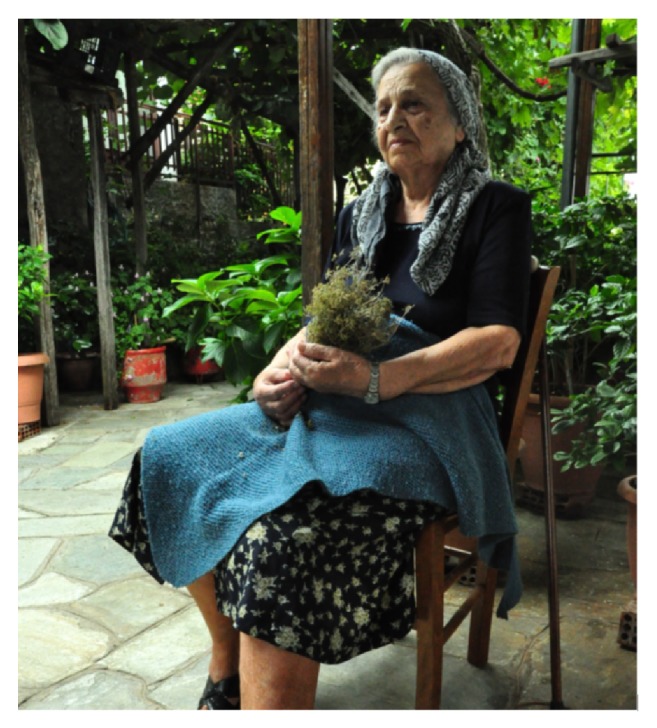
Informant interviewed during the ethnobotanical study.

For every informant we recorded personal information about age, gender, education level, profession, and population group. This distinction helped to note differences and similarities between citations based on different factors. The informants had personal experience in self-medication using herbs and had ethnobotanical knowledge because of family tradition or personal interest. Their age ranged between 24 and 94 years (mean = 59 years), educational levels included primary (30%), secondary (41%), and higher education (29%), jobs included employees (e.g., public employees, civil engineers), farmers, workers, and people involved in humanistic occupations (e.g., painter, teachers). All information was obtained after receiving an oral prior informed consent from the participants, according to the ISE (International Society of Ethnobiology) Code of Ethics. During the interviews, the informants were requested to indicate vernacular names of plants, parts of the plant used, association with other plants, folk uses, and preparation procedures. In many cases, data on specific recipes and their sources were included. Quite often the interviews took place in the village square or in the houses of the informants (Figure 2) where they also showed us traditional remedies that are currently used. Specimens of the plants were either given to us by the informants or collected from the wild, according to their instructions. The information collected refers to wild and cultivated species. The taxa were identified using standard Floras [24, 25, 28, 29]. Nomenclature is according to Dimopoulos et al. [30]. Voucher specimens are deposited at the Herbarium of the Aristotle University of Thessaloniki (TAU).

The data gathered on plant uses were organized in citations and each citation coincided with a single row in a database created using Microsoft Excel. Then data were analyzed and compared with several ethnobotanical references, primarily based on a search in Scopus database, using the search string “ethnobotany and Greece”. Following recent recommendations for reporting ethnobotanical field studies, primary data are presented in an unaltered form, allowing direct comparison between other similar researches [31]. In order to compare our results with the list of the most common medicinal plants used in Central Macedonia, in Table 1 we reported the species sold in the herbal market of the regional capital Thessaloniki [8]. Several ethnobotanical indices were adopted for interpreting the large quantity of information.

### 2.3. Quantitative Indices and Statistical Analysis

To estimate the use variability of the species, we adopted the Informant Consensus Factor [32], which was calculated for each medicinal category. This index was calculated as follows: number of citations in a subcategory (n_ur_) minus the number of taxa used in the same subcategory (n_t_), divided by the number of citations minus one [33, 34]:

(1) $$\begin{array}{c} F_{ic}=\frac{\left(n_{ur}-n_{t}\right)}{\left(n_{ur}-1\right)} \end{array}$$

The value of this factor ranges from 0 to 1. A high F_ic_ value indicates an agreement among the informants on the use of taxa within a medicinal subcategory. The F_ic_ reflects homogeneity of information provided by different informants.

The Fidelity Level index (FL) was also considered to indicate the informants' choice for a potential plant species to treat a given disease. It was calculated by the following formula [35]:

(2) $$\begin{array}{c} FL\;\left(\;\%\;\right)=\left(\;\frac{Np}{N}\;\right)*100 \end{array}$$

where Np is number of use reports for a given species reported to be used for a particular ailment category and N is total number of use reports cited for any given species.

Descriptive statistical analyses were carried out in R environment (vers. 3.4.0, R Core Team 2017) adopting a circlize package (vers. 0.4-2, [36]) and Venn Diagram package (vers. 1.6-19, [37]).

## 3. Results and Discussion

### 3.1. Medicinal Plants

Our results showed that 87 medicinal plant species belonging to 48 families are used in the study area to treat several ailments. The surveyed species are listed in Table 1, where plant families and species within each family are cited in alphabetical order. In this table, for each taxon reported, data on scientific name, family, local name, part of plant used, medicinal use, and number of citations and if they are wild or cultivated are included. In the last column, it is also reported if the species has been previously cited in the study of Hanlidou et al. [8] on the herbal market in Thessaloniki (representing ca. 55% of the taxa cited in our work).

Noteworthy, the knowledge regarding the medicinal plant use was rarely accompanied by preservation of linguistic diversity concerning the plant names. In fact, only 6 out of 87 taxa were cited also under their dialect names. Accordingly, also in previous works on ethnobotanical use of plants in folk medicine in Greece, dialect names were seldomly reported [6]. Lawrendiadis (1961), speaking about common names of medicinal plants in Greece, referred that they are usually related to the part of the body on which they have curative effects or to the disease against which they can be used. For example,* Tussilago farfara* was called “vychion” from the Greek word “vyx” (cough) or* Origanum dictamnus* “stomachochorton” from the Greek words “stomachos” (stomach) and “chorton” (herb). More recently, Hanlidou et al. [8], referring to medicinal plants sold in the Thessaloniki market, reported for most taxa both commercial names and names in Dioscurides.

#### 3.1.1. Most Cited Species

Data show that Lamiaceae (21%) and Asteraceae (13%) are the most represented families, followed by Rosaceae (6%). Among the taxa (species and subspecies) recorded, 23 are cultivated, either grown in the study area or purchased from the local market. However, most of the taxa (64) were collected from the wild. Among them, there is a Greek endemic taxon,* Satureja montana *subsp.* macedonica, *a member of the* S. montana* group restricted to Northern Central Greece, and six Balkan endemics (some of them extending to Italy or Anatolia), i.e.,* Achillea holosericea, Digitalis lanata, Helleborus odorus *subsp*. cyclophyllus, Sideritis scardica, Thymus sibthorpii, and Verbascum longifolium. *The remaining are more widespread plants, with a European (14 taxa), Mediterranean (16), Eurasiatic, or Cosmopolitan (23) distribution [30].

As shown in Table 2, the highest number of plants was used to treat respiratory and skin diseases followed by cardiovascular, gastrointestinal, genitourinary, nervous-sensorial, metabolic, and muscular-skeletal diseases. The most used parts of the plants were flowers and inflorescences (40%), followed by aerial parts (22%), leaves (16%), fruits and seeds (15%), and underground parts (8%). Principal methods of herbal preparations included infusion or decoction (76%), maceration in alcohol or oil (12%), used raw (7%), poultice (3%), and other (2%). The most cited species were* Hypericum perforatum*,* Matricaria chamomilla*, the endemic* Sideritis scardica, Tilia platyphyllos,* and* Sambucus nigra.*

About 48% of the medicinal plants mentioned by the informants had been previously reported to be sold in Thessaloniki market as traditional remedies [8]. Seventeen of the 22 most relevant species, with at least 8 citations and high Fidelity Level (Table 3), are among them. The traditional shops and the street markets of this city include interesting information mainly derived from the inherited knowledge of the herbal sellers. Lamiaceae, the most frequently recorded family both in our study area and in the market of Thessaloniki, includes the greatest number of species mentioned for treating digestive, nervous, and respiratory diseases [10].

In many cases, the most common species reported for Central Macedonia for medicinal purposes are in agreement with those cited from other regions of Balkan Peninsula. For example,* H. perforatum*,* M. chamomilla*,* S. nigra*,* R. canina*,* U. dioica,* and* C. monogyna *were frequently cited by informants from Bosnia-Herzegovina [1] and from south Kosovo [4]. In Northern Macedonia Albanian informants reported* H. tuberosum* as a remedy for improving heart contractility, in addition to the use as human food [3]. In the same area,* Sideritis scardica* was reported mainly for treatment of respiratory diseases.

#### 3.1.2. Plants Used for Respiratory Diseases

In our study* S. scardica* is one of the most important (FL = 100%) plants cited for respiratory diseases, followed by* Tilia platyphyllos*,* Sambucus nigra*,* Malva sylvestris, *and* Dactylorhiza sambucina*.* S. scardica* is an aromatic plant very popular in Greece, Bulgaria, Albania, and North Macedonia where it is largely used in local cuisines. The species, together with several related* Sideritis* species, is known as “mountain tea”, and in folk medicine of the Balkan countries, it is used for the preparation of decoctions mainly indicated to aid digestion, strengthen the immune system, and treat cold, flu, and allergies [6, 38]. It is also employed against shortness of breath, sinus congestion, and even pain and mild anxiety [5, 38, 39]. It is worth noting that tubers of* Dactylorhiza sambucina* (as well as other Orchidaceae species) are collected from the wild and used to prepare a beverage, called “salepi”, used as a cough remedy popular in several eastern Mediterranean countries [40]. Similar uses were reported for another Orchidacea,* Orchis morio* L., in south Kosovo where the tuber infusion is indicated as a remedy for influenza, stomach disorders, and wound healing [4].

#### 3.1.3. Plants Used for Skin Diseases

**Figure 3:**
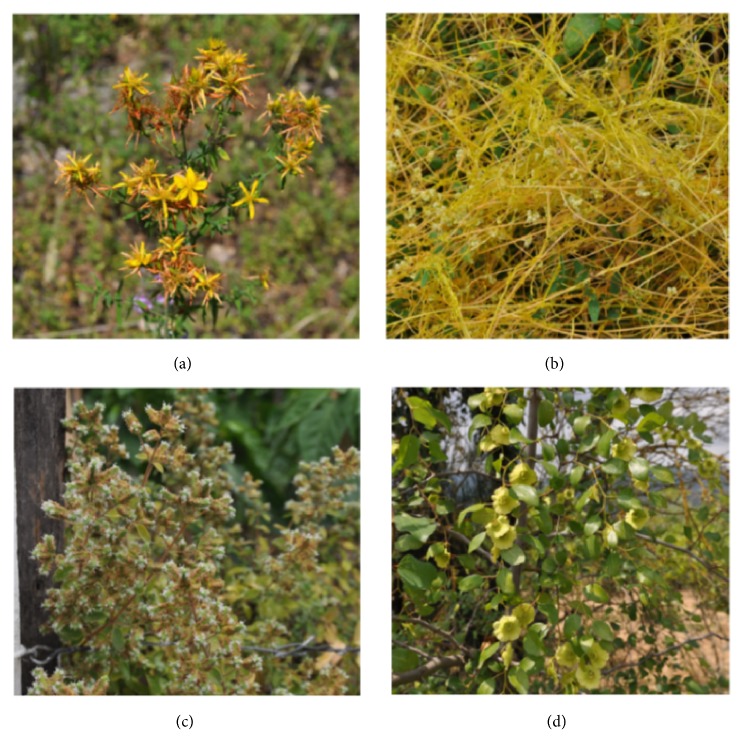
Plant species used to treat various ailments in the regions of Edessa and Naoussa: (a)* Hypericum perforatum,* (b)* Cuscuta campestris, *(c)* Origanum majorana, and *(d)* Paliurus spina-christi.*

Among plants used to treat skin diseases* Momordica charantia* shows the highest FL (94%), while* Hypericum perforatum* (Figure 3(a)) reaches the highest number of citations (44).* M. charantia* or bitter melon is a tropical vegetable extensively used in Indian folk medicine as a remedy for diabetes. In Ayurveda, the fruit is considered as tonic, stomachic, stimulant, emetic, antibilious, and laxative. In addition, the fruit juice and/or a leaf tea is employed for malaria, colic, sores, wounds, and other skin diseases [41].* M. charantia *fruit powder, in the form of an ointment (10% w/w dried powder in a simple ointment base), showed a statically significant response in terms of wound healing in rats [42]. In addition, recent studies showed that a* M. charantia* extract improves and accelerates the process of wound healing in rabbits in comparison with conventional creams used therapeutically [43]. It is a surprising fact that this tropical species has been incorporated in the traditional herbal medicine of the study area. In Macedonia we have previously reported the traditional preparation of an ointment made from the fragmented fruits of the plant that are immersed in olive oil and placed in the sun for 30–40 days. This ointment is indicated for the treatment of human and animal wounds [44]. The same use was referred by Turks living in south Kosovo, but in this case internal uses were also cited, including antidiabetic and anticancer [4].

A particular use of* Cuscuta campestris* (Figure 3(b)) was reported as a topic remedy against bee sting. Some of the aromatic plants used for the treatment of gastrointestinal problems are* Origanum majorana* (Figure 3(c)),* Matricaria chamomilla, *and* Mentha spicata*. It is interesting to note that this latter species is included within plant taxa sold as “mint” in the market of Thessaloniki, where it is mostly recommended for common cold and cough [9].* O. majorana* is widely used to treat colds and rhinitis [45, 46], but it is also quoted for its antiulcer effect [47]. In addition,* Castanea sativa* catkins are widely used as a decoction for the treatment of diarrhea.

#### 3.1.4. Plants Used for Cardiovascular Diseases

Two species were found to be the most quoted (FL = 100%) for the treatment of cardiovascular diseases:* Crataegus monogyna* and* Loranthus europaeus*.* C. monogyna* is popularly known for its cardioprotective action reducing cardiovascular risk factors, such as hypertension and hypercholesterolemia [48, 49]. In Macedonia this species is used as an infusion prepared with flowers, fruits, or leaves, but also as an alcoholic extract prepared from the fruits.* L. europaeus* (yellow-berried mistletoe) is a hemiparasite plant usually found on the branches of trees. The popular use of this plant for the treatment of cardiovascular problems has also been reported in Bosnia and Herzegovina [1]. In addition, a recent study on the ethnobotany of mistletoes species also cited the frequent use of this plant to treat cardiovascular disorders [50].

#### 3.1.5. Plants Used for Nervous Diseases

The use of* Melissa officinalis* and* Valeriana officinalis* to cure nervous system problems is well known [51, 52], while that of* Salvia officinalis* is less common. In the traditional medicine of many European countries this species has been used mainly to treat mild dyspepsia, excessive sweating, and throat and skin inflammations [53], but the use in age-related brain disorders has also been reported [54]. In addition, some informants reported an uncommon use of* Taraxacum officinale* for treating nervous diseases, i.e., headache and insomnia. This use has been previously reported in an ethnobotanical survey of Zagori, Epirus, Greece [6].

#### 3.1.6. Plants Used for Genitourinary Diseases

Different plant parts of a large variety of species are used for genitourinary diseases, as diuretic and in prevention and treatment of prostatitis, mainly in the form of a decoction. Among them, the most quoted are* Micromeria juliana *(8 citations, FL.= 100%),* Paliurus spina-christi* (10 citations, FL= 91%; Figure 3(d)), and* Achillea* spp.

#### 3.1.7. Plants Used for Musculoskeletal Diseases

A large number of species are used for musculoskeletal diseases, in particular against joint pains and rheumatism, such as* Equisetum arvense* (aerial parts),* Arctium lappa* (root),* Cercis siliquastrum* and* Robinia pseudoacacia* (flowers). The alcoholic extract of the sliced root of* Tamus communis* is also employed to treat muscular pain and inflammation. This species shows toxicity due to its content of calcium oxalates, saponins, tannins, and other substances; nevertheless, its anti-inflammatory and analgesic properties are well known [55] and its use in the treatment of rheumatisms has also been reported in the traditional medicine of Turkey [56] and Portugal [57].

#### 3.1.8. Plants Used for Metabolic Diseases

The most quoted plant for the treatment of metabolic diseases is* Rosa canina *(17 citations, FL = 85%), followed by* Urtica dioica* and* Silybum marianum*. In particular,* R. canina *is used as a decoction of fruits and flowers, syrup of boiled fresh fruits but also eaten raw as an immunostimulant, and for the prevention of the cardiovascular diseases.

### 3.2. Plants with Unusual Medicinal Uses or Used Only by a Single Population Group

Some medicinal uses of plants seem to be particularly interesting because they have been infrequently or never reported. In addition, some of these uses concern toxic plants and rare or endemic species.* Cuscuta campestris* stems (Figure 3(b)) are applied topically against bee stings; Dopioi informants have exclusively reported this use. There are only a few ethnobotanical uses that have been detailed for this species: as purgative and against constipation in Saudi Arabia [58] and for its analgesic effect in the treatment of rheumatisms and headaches in Nepal [59].

In the present study, the use of a toxic species,* Cynoglossum creticum, *which contains pyrrolizidine alkaloids, has been cited [60]. The fruits of this plant have been reported to be eaten to treat a skin disease called* “myrmecia”* (Greek “myrmigkia”= ant), a word used allover Greece for the common skin warts caused by HPV (Human Papilloma Virus). The disease is characterized by the presence of small skin warts apparently similar to anthills. In the traditional medicine of India the aerial portion of* Cynoglossum* spp. is used against wounds, burns, ear infections, and cough for its antibacterial properties [61] and against fungal skin infections [62]. In Sardinia the root is used to prepare an emollient cream against burns [63], while leaves are directly applied as a cicatrizant or as a poultice to treat eczema [64]. In this study only informants from the group “others” have cited this particular use of* C. creticum* for the treatment of* myrmecia*.

*Satureja montana* subsp.* macedonica*, in addition to the common use of the flower infusion for the treatment of flu and cough, is also used in the study area by Pontians informants to relieve tinnitus and improve hearing. Generally,* Satureja* spp. have been used since ancient times as flavorings for food and for the treatment of various diseases; their essential oils have been documented for antimicrobial, antidiarrheal, fungicidal, and antioxidant activities [65]. However, the specific use for tinnitus treatment and for hearing improvement is particularly unusual and, to our knowledge, has never been previously reported.

*Hyssopus officinalis* is an aromatic plant commonly used in the traditional medicine of the Balkan Peninsula for its antiseptic, carminative, and spasmolytic properties [66]. The aerial parts of the species are used against chronic bronchitis and asthma [67, 68]. Notably, the use of the decoction from the aerial parts as an analgesic to treat headache, cited in the present study, has never been previously reported.

*Helianthus tuberosus* is a naturalized species, native to North America, which was introduced in Europe during the XVII century. It was used at first as animal feed and subsequently as human food. Its tuber contains inulin as its main polysaccharide and is therefore indicated in the diet of diabetics. In the Carpathian basin it is also used in the treatment of asthma and heart problems [69]. Although it had not been highly reported in traditional medicine of the Mediterranean region, in the present survey, the decoction of the fragmented tuber was indicated against constipation. Similar uses have been described in Austria [70]. Also in south Kosovo the use of fresh tubers of* H. tuberosum* as human food was recently reported [4].

Finally,* Equisetum arvensis* (10 citations) and* Robinia pseudoacacia* (4 citations) were used by Dopioi only, while* Castanea sativa *(6 citations) by the group “others”. Moreover 7 out of 8 citations concerning* Satureja montana* came from Pontians.

### 3.3. Quantitative Analyses

#### 3.3.1. Traditional Ethnobotanical Knowledge among Different Population Groups

**Figure 4:**
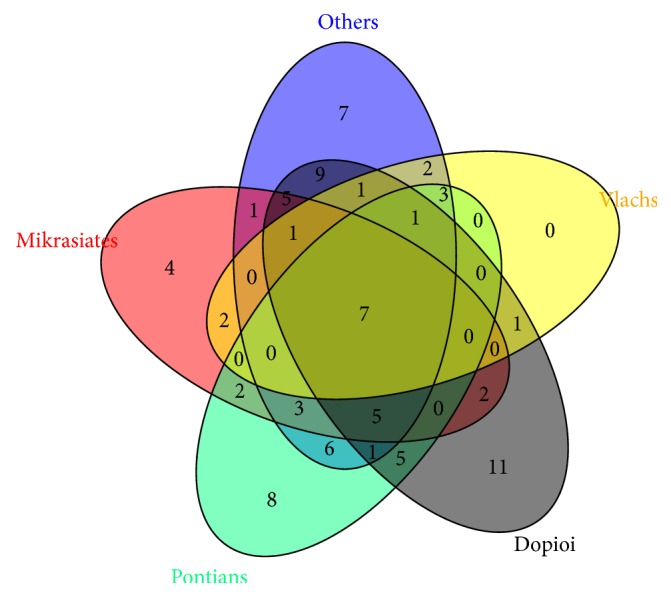
Venn diagram representing an overlap of Traditional Ethnobotanical Knowledge of the 5 population groups. Numbers are the species in common for each combination of overlapping (including nonoverlapping subsets, i.e., species reported by a single population group).

The group “others” (i.e., informants who come from various parts of Greece) and Dopioi reported the highest number of species (53), followed by Pontians (42) and Mikrasiates (23 species). Vlach informants only cited 10 species. Only 7 species (*Crataegus monogyna*,* Hypericum perforatum*,* Matricaria chamomilla*,* Rosa canina*,* Sambucus nigra*,* Sideritis scardica,* and* Tilia platyphyllos*) were commonly reported by all population groups (Figure 4), whereas 30 out of 87 taxa (34%) were exclusively mentioned by a single group: 11 by Dopioi, 8 by Pontians, 7 by the group “others”, and 4 by Mikrasiates. The species reported by Mikrasiates and Vlachs were mostly or fully shared with one or more of the groups. Notably, only 12% of taxa were referred with more than one name and only 6% were known with different dialectal names by different population groups (Table 1). It was demonstrated that in different Balkan areas that share similar flora but have different cultural or linguistic heritage, medicinal plants are used in very distinct ways [71–73]. For example, Mustafa et al. [4] found that Albanian, Bosniak/Gorani, and Turkish communities of Kosovan villages shared 22% of the taxa used for food and medicine, suggesting a hybrid character of the Kosovar plant knowledge. However, 42% of the plant species were only cited by a single ethnic group.

#### 3.3.2. Plant Knowledge according to Sociologic Variables

**Figure 5:**
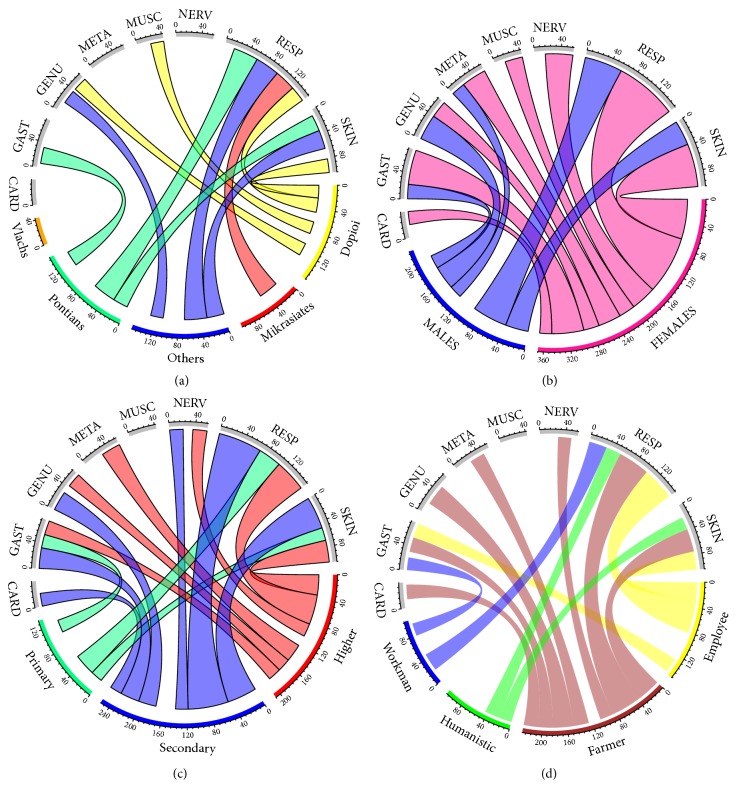
Circular plots showing the relationships among referred disease categories and the informants grouped on the basis of different social factors: (a) origin; (b) gender; (c) education; (d) job. Color bands summarize main relationships (> 20% of the total information) between each disease category and population groups. Numbers below the external segments in the graphs represent the total numbers of citations for each disease category and population group (e.g., in Figure 5(a), 40 citations were overall reported for plants used as a remedy for muscular-skeletal diseases, whereas a total of 132 citations were provided by Dopioi).

The diagrams in Figure 5 report the most important remedies-informant groups associations (> 20% of the total information reported for each disease category) observed, as a function of the main social categorizations of informants interviewed in our survey. When analyzing the distribution of disease categories in relation to population groups, some significant differences have been observed (Figure 5(a)). The knowledge of less numerous groups (Vlachs and Mikrasiates) was equally distributed among disease categories, but less relevant with respect to that provided by the other groups. Plant species used against skin and respiratory diseases were highly cited by other informant groups, such as Pontians, Dopioi, and “others”. For these latter about 50% of the information is related to these diseases. Dopioi showed important contribution in four remedy categories, being highly prevalent in the citations of treatments for muscular disorders. Similarly, the information delivered by Pontians represented nearly half of the total knowledge about the treatments for gastrointestinal diseases.

Although both male and female informants cited species that are used for different healing purposes, the ethnobotanical knowledge of females was more equally distributed in all use categories, whereas men mainly reported information on respiratory, genitourinary, and skin diseases (Figure 5(b)).

Remedies for respiratory diseases were highly cited by informants, independently from their educational level, whereas the knowledge of medicinal plants used for treating nervous, skin, and gastrointestinal diseases was mainly reported by informants with a secondary level of education. Graduate informants had a major degree of knowledge about the treatment of metabolic disorders (Figure 5(c)).

Independently from their job, many informants reported treatments of respiratory diseases (Figure 5(d)). Moreover, in contrast with other job groups, the information reported by farmers was largely distributed among nearly all disease categories. Unexpectedly, no relevant differences were observed in relation to the age of the informants: both younger and older groups equally cited plant remedies for most of the diseases (data not shown). However, some associations were observed: persons < 40 years old frequently cited remedies for skin diseases, whereas plants used for treating genitourinary disorders were mainly cited by informants between 60 and 70 years old.

## 4. Conclusions

Our study highlights the importance of reporting the TEK typical of areas of Europe that have until now been poorly investigated. In fact, despite the large number of papers dealing with ethnobotany in the Balkans and southeastern Europe [74, 75], Greece is still scarcely explored from an ethnobotanical point of view although it is characterized by a high floristic diversity. This is particularly true for regions like Central Macedonia where different population groups live together, maintaining most of their traditions. Information on species used in folk medicine of this region showed that most of them were wild and cultivated plants well known in the European ethnobotany for their healthcare and curative properties. Nevertheless some unusual uses were found, in particular concerning several toxic plants and rare or endemic species. Despite growing erosion of existing European tradition ethnobotanical knowledge, population groups in this region maintain some exclusive folk remedies such as the use of* Cuscuta campestris* against bee stings reported only by Dopioi informants and that of* Satureja montana* subsp.* macedonica* cited by Pontians to relieve tinnitus and improve hearing. Such local knowledge is culturally significant and can provide information for developing future researches and promoting ethnopharmacological advances. When taking in account differences among the studied population groups, only 7 plant species were commonly reported by all groups, whereas 34% of plants were exclusively mentioned by a single group. This observation supports the idea that some differences are maintained among population groups, although the knowledge on the medicinal plant use is not accompanied by preservation of linguistic diversity concerning the plant names. Furthermore our analysis of plant knowledge according to sociologic variables contributes to a better understanding of factors that affect changes in plant uses and perceptions in different sociocultural contexts.

## Figures and Tables

**Table 1:** Ethnobotanical uses of plants in the regions of Edessa and Naoussa (Central Macedonia, Greece).

Families/Species	Local names	Parts used	Use categories, preparation, and ailment treated	No. citations per population group					No. of total reports	WS/CS	Sold in Thessaloniki market [8] *∗*
				OTH	MI	PO	DO	VL			
**Adoxaceae**											
*Sambucus nigra* L. (TAU 60500)	Zampouko	Inf	MED-RES: decoction against inflammation of the respiratory tract, cough or as expectorant, (also combined with Le. of *Alcea rosea* or with Se of* Linum usitatissimum*, Ap of *Verbascum longifolium* and *Thymus vulgaris*)	5	1	3	7	2	21	W	+
			MED-SKI: stems' bark with wax and olive oil applied on cuts, bruises, burns, and wounds	1	0	2	1	0			

**Amaryllidaceae**											
*Allium cepa *L.	Kremidi	Bu	MED-SKI: used as cataplasm with salt or wine or olive oil against skin burns, bruises, and edemas	0	2	0	1	0	3	C	+
*Allium sativum *L.	Skordo	Bu	MED-CAR: eaten raw or cooked to prevent and treat hypertension	0	0	1	3	0	4	C	+

**Anacardiaceae**											
*Pistacia lentiscus *L. var. chia	Masticha	Re	MED-GAS: chewed against gastric ulcers and ailments of the digestive system	0	0	1	0	0	1	C	+

**Apiaceae**											
*Apium graveolens *L.	Selino	Le	MED-CAR: decoction against hypertension	1	0	0	0	0	2	C	
			MED-RES: boiled with milk to treat the symptoms of common cold	1	0	0	0	0			

**Aquifoliaceae**											
*Ilex aquifolium *L. (TAU 60501)	Ou	Le	MED-MUS: decoction to treat joint and muscular pains	0	0	0	1	0	1	W	

**Araceae**											
*Arum italicum *Mill. (TAU 60502)	Drakontia/ Smiina bilka (DO)	Le, Ro	MED-RES: decoction against cough, bronchial catarrh, and sore throat	0	0	1	1	0	2	W	
*Arum maculatum *L. (TAU 60503)	Drakontia	Le, Ro	MED-RES: decoction against cough, bronchial catarrh, and sore throat	0	0	1	1	0	2	W	

**Araliaceae**											
*Hedera helix* L. (TAU 60504)	Kissos	Le	MED-RES: decoction against cough	2	0	0	1	0	3	W	+

**Asparagaceae**											
*Ruscus aculeatus *L. (TAU 60505)	Agathi	Ap	MED-GEN: decoction for the treatment and prevention of prostatitis and as diuretic (combined in the decoction with Fr. of* Paliurus spina-christi*)	0	0	0	3	0	3	W	+

**Asphodelaceae**											
*Aloe vera *(L.) Burm. f.	Aloi	Le	MED-SKI: parenchymatic gel used as poultice applied on wounds, sunburns, and insect bites	0	0	0	1	0	1	C	

**Asteraceae**											
*Achillea holosericea* Sm. (TAU 60506)	Kitrini Achillia	Inf	MED-RES: decoction against inflammation of the respiratory tract and cough or as expectorant	0	0	0	0	2	4	W	
			MED-GEN: decoction against cystitis	0	0	0	0	1			
			MED-SKI: water of maceration as cataplasm against hemorrhoids	1	0	0	0	0			
*Achillea millefolium* L. (TAU 60507)	Lefki Achillia	Inf	MED-RES: decoction against inflammation of the respiratory tract and cough	0	0	3	0	2	17	W	+
			MED-GEN: decoction against cystitis, urogenital inflammation, and regulation of menstrual cycle (with *Capsella bursa-pastoris*, cinnamon bark and orange peel)	4	0	5	0	1			
			MED-SKI: water of maceration as cataplasm against hemorrhoids; used fresh as cataplasm against acne	1	0	1	0	0			
*Arctium lappa* L. (TAU 60508)	Arktio	Ro	FOOD: eaten raw as depurative	1	0	2	0	0	11	W	+
		Le	MED-MUS: decoction for joint pain and inflammation	2	1	2	0	0			
		Le	MED-SKI: decoction used as poultice applied on wounds and furuncles	1	0	2	0	0			
*Cirsium eriophorum* (L.) Scop. (TAU 60509)	Kirsio	Fl	MED-MET: decoction for detoxification and treatment of liver diseases	0	2	0	0	0	2	W	
*Helianthus tuberosus *L. (TAU 60510)	Kolokasi	Tu	FOOD: eaten boiled as salad to improve intestinal function	0	0	3	0	0	6	C	
			MED-GAS: decoction against constipation	0	0	3	0	0			
*Lactuca serriola *L. (TAU 60511)	Agriomarulo	Le, Fl	MED-MET: decoction against hypercholesterolemia	2	0	0	1	0	3	W	
*Matricaria chamomilla *L. (TAU 60512)	Chamomili	Inf	MED-NER: decoction or infusion against insomnia and otitis	1	6	1	5	1	33	W	+
			MED-GAS: decoction against abdominal pain	3	3	4	1	0			
			MED-GEN: washing to treat vaginitis	3	0	1	0	0			
			MED-RES: oily extract or decoction against cough		1						
			MED-SKI: oily extract applied for wound healing	3	0	0	0	0			
*Pulicaria dysenterica *(L.) Bernh.	Pulicaria	Ap, Fl	MED-GAS: decoction for constipation and intestinal problems	1	0	0	0	0	1	W	
*Silybum marianum* (L.) Gaertn. (TAU 60513)	Gaiduragatho	Fl, Fr	MED-MET: decoction for detoxification and treatment of liver diseases	3	0	0	2	0	5	W	+
*Taraxacum* sp. (TAU 60514)	Taraxaco	Ap	MED-NER: decoction against headache and insomnia	1	0	1	0	1	4	W	+
			MED-MET: to decrease triglyceride and cholesterol levels	1	0	0	0	0			
*Tussilago farfara *L. (TAU 60515)	Vihio/ Flomos	Ap	MED-RES: decoction against cough and inflammation of the respiratory tract	2	0	0	1	0	3	W	+

**Boraginaceae**											
*Cynoglossum creticum *Mill.	Kynoglosso/	Fr	MED-SKI: eaten triturated for the treatment of warts	4	0	0	0	0	4	W	
	Aritsovotano										

**Caprifoliaceae**											
*Valeriana officinalis *L. (TAU 60516)	Valeriana	Ro	MED-NER: tincture against insomnia and as calmative (combined with *Capsella bursa-pastoris*)	1	0	4	0	0	5	W	+

**Caryophyllaceae**											
*Saponaria officinalis* L. (TAU 60517)	Sapunochorto	Fl	MED-SKI: cataplasm applied on skin against eczema and dermatitis	2	0	0	1	0	3	W	+

**Convolvulaceae**											
*Cuscuta campestris* Yunck. (TAU 60518)	Kitrino parasito	Ap	MED-SKI: stems of the aerial part rubbed on bee stings	0	0	0	3	0	3	W	

**Cornaceae**											
*Cornus spp.*	Krana	Fr	MED-GAS: alcoholic extract drunk against abdominal pain		1				1	W	

**Cucurbitaceae**											
*Cucurbita pepo *L.	Kolokithi	Se	MED-GEN: oily extract drunk to cure and prevent prostatitis	0	0	0	1	0	2	C	
			MED-MUS: oily extract topically applied against joint pain	0	0	0	1	0			
*Momordica charantia* L. (TAU 60519)	Kanturi	Fr	MED-SKI: oily extract against wounds, burns, calluses; drunk against gastric ulcers.	5	2	0	6	1	30	C	
			VET: oily extract used on goats and pigs to cicatrize wounds	5	2	0	6	0			

**Cupressaceae**											
*Juniperus communis *L. (TAU 60520)	Mavros Kedros	Ga	MED-CAR: eaten raw or used as flavoring for meat to prevent cardiovascular diseases	0	0	2	0	0	2	W	
*Juniperus oxycedrus* L. (TAU 60521)	Kedros	Ga	FOOD-MED: flavoring for meat and digestive	0	0	1	1	0	2	W	+

**Dennstaedtiaceae**											
*Pteridium aquilinum *(L.) Kuhn (TAU 60522)	Fteri	Le	DOM: wrap up raw meat to keep it fresh	0	1	0	4	0	7	W	
			MED-GEN: decoction as diuretic and to remove kidney stones		1			1			

**Dioscoreaceae**											
*Tamus communis* L.	Riza tou Adam	Ro	MED-MUS: alcoholic extract of sliced root (forms a cream) against muscular pain and inflammation	1	0	2	0	0	3	W	

**Equisetaceae**											
*Equisetum arvense *L. (TAU 60523)	Ekuiseto/ Polikombi/Ura tu alogu	Ap	MED-GEN: decoction against urogenital diseases and for the treatment of prostatitis as diuretic	0	0	0	10	0	10	W	+

**Ericaceae**											
*Vaccinium myrtillus *L. (TAU 60524)	Mirtillo	Fr	MED-NER: eaten raw to improve vision	0	0	2	0	0	3	W	+
			MED-SKI: pulped against gums' inflammation		1						

**Fabaceae **											
*Robinia pseudoacacia* L. (TAU 60525)	Akakia	Fl	MED-MUS: decoction against joint pains and rheumatisms (also combined with Fl. of *Cercis siliquastrum*)	0	0	0	5	0	5	W	
*Cercis siliquastrum* L. (TAU 60526)	Kutsupia	Fl	MED-MUS: decoction against joint pains and rheumatisms (also combined with Fl. of *Robinia pseudacacia* and Le. of *Alcea rosea*)	0	0	0	7	0	7	W	

**Fagaceae**											
*Castanea sativa* Mill. (TAU 60527)	Kastania	Fl	MED-GAS: decoction against diarrhea	6	0	0	0	0	6	C	+

**Grossulariaceae**											
*Ribes uva-crispa *L. (TAU 60528)	Fragostaphilo	Ap	MED-MET: decoction or tincture to increase iron levels and as tonic	1	0	2	0	0	3	W	

**Hypericaceae**											
*Hypericum perforatum* L. (TAU 60529)	Valsamochorto/ Rumana/ Spathochorto	Inf	MED-SKI: oily or alcoholic extract applied on wounds, burns; mixed with wax and Re. of *Pistacia lentiscus* var. *chia* and applied on deep sores	8	7	14	6	9	65	W	+
			MED-MUS: as massage to alleviate arthritis symptoms and joint pain	0	1	3	0	0			
			MED-GAS: drunk to treat gastric ulcers and gastrointestinal disturbs (1 spoon/day)	1	4	6	1	3			
			MED-NER: decoction against insomnia and as antidepressant	0	0	1	1	0			

**Iridaceae**											
*Crocus sativus *L.	Krokos	Fl	MED-MET: water of maceration of stigmas as immunostimulant or for the prevention of common colds and flu	0	0	1	0	0	1	C	+

**Lamiaceae**											
*Hyssopus officinalis* L. (TAU 60530)	Issopos	Ap	MED-NER: decoction against headache	1	0	2	0	0	3	C	
*Lavandula angustifolia *Mill. (TAU 60531)	Levanta	Inf	MED-NER: decoction as bland sedative	1	1	1	0	0	6	C	+
			MED-CAR: against hypertension if combined with Le. of *Mentha spicata*	1	1	1	0	0			
*Melissa officinalis*	Melissochorto	Le	MED-NER: decoction or infusion as sedative; used as decoction in combination with Ap. of* Crataegus monogyna* Jacq. for the prevention of cardiovascular diseases	4	0	0	2	0	6	W	+
subsp. *altissima* (Sm.) Arcang. (TAU 60532)											
*Mentha spicata *L. (TAU 60533)	Menta	Le, Ap	MED-MET: decoction or infusion against hypercholesterolemia	1	0	0	4	0	18	W	+
			MED-CAR: cardiovascular problems and hypertension	1	0	0	4	0			
			MED-GAS: decoction in combination with Inf. of *Hypericum perforatum* for the treatment of gastric ulcers, nausea and flatulence	1	2	1	4	0			
*Mentha spp.*	Menta/Dyosmos	Le	MED-GAS: decoction against nausea and flatulence		2			1	6	W	
			MED-RES: decoction against inflammation of the respiratory tract and cough		2						
					1						
*Micromeria juliana *(L.) Rchb. (TAU 60534)	Kiparissaki	Ap	MED-GEN: decoction as diuretic and for the prevention and treatment of prostatitis (also in combination with Se of* Rosa canina* and Ap. of* Equisetum arvense*)	1	1	6	0	0	8	W	+
*Ocimum basilicum *L. (TAU 60535)	Vasilikos	Le	MED-NER: used fresh as decoction or infusion against insomnia and to improve memory and concentration	0	1	0	2	0	3	C	+
*Origanum dictamnus *L.	Diktamo	Ap	MED-RES: decoction against cough and symptoms of common cold		1				1	C	+
*Origanum majorana *L. (TAU 60536)	Matzurana	Ap	MED-GAS: decoction as digestive, against nausea and gastric disturbs	1	2	3	1	0	8	C	+
			MED-GEN: decoction against menstrual pain		1						
*Origanum vulgare *	Rigani	Ap	MED-MET: decoction or water of maceration as blood depurative	0	0	0	2	0	3	W	+
subsp. *hirtum* (Link) Ietswaart (TAU 60537)											
			MED-GAS: eaten raw to improve digestion					1			
*Origanum vulgare *	Tourkiko tsai	Inf	MED-RES: decoction with honey and butter against allergic cough	2	0	0	0	0	2	W	
subsp. *viridulum* (Martrin-Donos) Nyman (TAU 60538)											
*Rosmarinus officinalis *L. (TAU 60539)	Dentrolivano	Ap	MED-MET: decoction against hypercholesterolemia	0	4	0	0	0	4	C	+
*Salvia officinalis *L. (TAU 60540)	Faskomilo	Le	MED-NER: decoction or water of maceration against insomnia and anxiety (in combination with Inf. of* Aloysia citrodora*)	2	1	0	3	0	6	W	
*Satureja montana *subsp. *macedonica* (Formànek) Baden (TAU 60541)	Thymari/ Thympiron (PO)	Inf	MED-RES: decoction against inflammation of the respiratory tract and for cough	0	0	4	0	0	8	W	
			MED-MET: decoction against hypercholesterolemia	0	1	0	0	0			
			MED-NER: decoction to alleviate symptoms of tinnitus	0	0	3	0	0			
*Sideritis montana *	Aspro tsai	Inf, Ap	MED-RES: decoction against inflammation of the respiratory tract and cough	2	0	1	0	2	5	W	
subsp. *remota* (d'Uvr.) P.W. Ball (TAU 60542)											
*Sideritis scardica *Griseb. (TAU 60543)	Tsai tu vunu	Inf, Ap	MED-RES: decoction against inflammation of the respiratory tract and cough	6	11	7	2	4	30	W	+
*Teucrium capitatum *L. (TAU 60544)	Tefkrio	Fl	MED-GAS: decoction against abdominal pain	0	0	1	2	0	3	W	
*Thymus sibthorpii *Benth. (TAU 60545)	Materina	Inf, Le	MED-GAS: decoction against abdominal pain or gastrointestinal disturbs	3	0	0	0	1	4	W	

**Linaceae**											
*Linum usitatissimum *L.	Linari	Se	MED-GAS, decoction against constipation	0	0	3	0	0	13	C	+
			MED-RES: decoction or infusion as expectorant or to alleviate cough (also in combination with Inf. of* Sambucus nigra* and Ap. of *Verbascum longifolium*. and *Thymus vulgaris*)	1	3	5	0	0			
			MED-SKI: as cataplasm to treat wounds and furuncles		1						

**Loranthaceae**											
*Loranthus europaeus *Jacq. (TAU 60546)	Parasito	Ap	MED-CAR: decoction of twigs to treat hypertension, varicose veins and to improve blood circulation	2	0	0	5	0	7	W	

**Malvaceae**											
*Alcea rosea *L. (TAU 60547)	Althea	Le	MED-RES: decoction or infusion against cough in combination with Inf. of* Sambucus nigra*; decoction against rheumatisms and musculoskeletal pain in combination with Fl. of *Robinia pseudacacia* and of *Cercis siliquastrum*	1	0	0	3	0	4	W	
*Malva sylvestris* L. (TAU 60548)	Molocha	Fl	MED-RES: decoction or infusion as expectorant and against cough	6	4	2	1	0	14	W	+
*Tilia platyphyllos* Scop. (TAU 60549)	Tilios/Flamuri	Inf	MED-GAS: decoction against intestinal pain	0	2	2	1	0	26	W	+
			MED-RES: decoction for cough and bronchial catarrh	3	5	6	6	1			
			MED-NER: decoction as sedative	0	0	0	1	0			

**Oleaceae**											
*Fraxinus ornus *L. (TAU 60550)	Fraksos	Ap	VET: bark of stems into chickens' water to prevent their illnesses	0	0	1	1	0	2	W	+

**Onagraceae**											
*Epilobium angustifolium *L. (TAU 60551)	Epilovio	Le	MED-GEN: decoction to treat and prevent prostatitis or as diuretic	3	1	0	1	1	6	W	
*Epilobium parviflorum* Schreb.	Epilovio	Le	MED-GEN: decoction to treat and prevent prostatitis and cystitis or as diuretic	1	0	0	0	0	1	W	

**Orchidaceae**											
*Dactylorhiza sambucina* (L.) Soó (TAU 60552)	Salepi	Bu	MED-RES: decoction as expectorant against cough and common cold	5	0	2	0	1	8	W	

**Papaveraceae**											
*Papaver rhoeas *L. (TAU 60553)	Paparuna	Se	MED-NER: infusion or decoction as sedative	0	0	2	0	0	2	W	

**Passifloraceae**											
*Passiflora *sp. (TAU 60554)	Passiflora	Fl	MED-NER: decoction as sedative	0	0	0	2	0	2	C	+

**Piperaceae**											
*Piper nigrum *L.	Piperi	Se	MED-RES: alcoholic extract used as cataplasm against cough		1				1	W	+

**Plantaginaceae**											
*Plantago media *L. (TAU 60555)	Pentanevro/ Tigavits (DO)	Le	MED-SKI: used fresh, rubbed on wounds, furuncles and hemorrhoids	3	1	1	3	0	10	W	
			MED-GEN: decoction or eaten as salad to treat or prevent urogenital ailments (prostatitis) and improve kidneys' function	2	0	0	0	0			
*Digitalis lanata *Ehrh. (TAU 60556)	Digitalis	Le	MED-CAR: decoction to improve blood circulation and prevent cardiovascular diseases	1	0	0	0	0	1	W	

**Poaceae**											
*Elytrigia repens *(L.) Nevski (syn. *Agropyron repens *(L.) P. Beauv.)	Agriada	Ap	MED-MUS: decoction or infusion against joint and muscular pains	1	1	0	0	0	2	W	
*Zea mays *L.	Kalampoki	StyFl	MED-NER: decoction or water of maceration as sedative	1	1	0	1	0	6	W	+
			MED-GEN: decoction in combination with fruit stalksof *Prunus avium* as diuretic or for the prevention and treatment of urogenital disturbs	2	1	0	0	0			

**Portulacaceae**											
*Portulaca oleracea* L. (TAU 60557)	Glistrida/ Adrakla	Ap	MED-MET: eaten raw or boiled against hypercholesterolemia	0	0	1	1	0	6	W	
			MED-NER: eaten raw against toothache and to remove tartar	0	0	0	1	0			
			MED-CAR: eaten raw to prevent cardiovascular diseases, hypertension, obstructed arteries	0	0	1	2	0			

**Ranunculaceae**											
*Helleborus odorus* subsp*. cyclophyllus* (A. Braun) Maire & Petitm. (TAU 60558)	Elevoros/ Spres (DO)	Ro	MED-MUS: decoction of fragmented root (1 part of root in 2 liters of water) to treat joint pain; toxic plant	0	0	0	2	0	4	W	
			VET: decoction of fragmented root with salt to treat joint pain in goats, cows and pigs	0	0	0	2	0			

**Rhamnaceae**											
*Paliurus spina-christi* Mill. (TAU 60559)	Paliuri/ Fluri	Fr	MED-GEN,: decoction for urinary tract and urogenital diseases and the treatment of prostatitis as diuretic	3	0	1	6	0	11	W	+
			MED-MET: decoction against hypercholesterolemia	0	1	0	0	0			

**Rosaceae**											
*Agrimonia eupatoria* L. (TAU 60560)	Agrimonio	Ap	MED-SKI: infusion applied as cataplasm for the cicatrization of wounds	1	0	2	0	0	3	W	+
*Crataegus monogyna* Jacq. (TAU 60561)	Krategos	Ap, (Fl, Fr, LeSh)	MED-CAR: decoction (Ap) or alcoholic extract (Fr) for the prevention of cardiovascular diseases and for the treatment of hypertension	1	4	2	3	3	13	W	+
*Prunus avium* L. (L.) (TAU 60562)	Kerasia	FrPed, Se	MED-MUS: decoction against arthritis and joint pain (in combination with StyFl. of* Zea mays*); as diuretic	1	0	0	1	1	3	C	+
*Rosa canina* L. (TAU 60563)	Agriotriantafilia/ Kynorodo/ Masura (PO)	Fl,	FOOD: marmalade; tonic liquor (made of petals)	0	0	0	2	0	24	W	+
		Fr									
		Se	MED-MUS: against rheumatisms in combination with Fl. of* Cercis siliquastrum*; to treat osteoarthritis; eaten pulverized against arthritis pain	0	1	0	1	0			
		Fr									
		Fr	MED-GEN: decoction helps to remove kidney stones, against prostatitis in combination with StyFl. of* Zea mays* and Ap. of* Micromeria juliana*	0	0	1	0	0			
		Fl, Fr	MED-MET: eaten raw as antioxidant, for the prevention of cardiovascular diseases; decoction, syrup, marmalade or liquor as immunostimulant (to prevent cold and flu)	3	3	4	7	2			
*Rosa x damascena* Herrm. (TAU 60564)	Palia triantafyllia	Fl	MED-MUS: oily extract of petals applied to treat musculoskeletal pain/also used to treat children's joint pain while they grow up	0	0	0	2	0	2	C	

**Rutaceae**											
*Citrus limon *(L.) Osbeck	Lemoni	Le	MED-MET: decoction against hypercholesterolemia	0	0	2	0	0	2	C	

**Scrophulariaceae**											
*Verbascum longifolium* Ten. (TAU 60565)	Verbasco/ Lupusiou (VL)	Ap	MED-RES: decoction to treat cough	3	0	3	1	0	10	W	
		Ap	MED-GEN: decoction to treat and prevent prostatitis	0	0	2	0	0			
		Le	MED-GAS: decoction against abdominal pain	0	0	2	0	0			

**Solanaceae**											
*Alkekengi officinarum* Moench. (syn. *Physalis alkekengi* L.) (TAU 60566)	Fanaraki	Fr	MED-MET: eaten raw as immunostimulant and tonic	1	0	0	1	0	2	W	
*Nicotiana tabacum* L. (TAU 60567)	Kapnos	Le	MED-SKI: used fresh, applied on wounds as cicatrizing, hemostatic, disinfectant	0	0	2	0	0	2	C	

***Urticaceae***											
*Urtica dioica *L. (TAU 60568)	Tsouknida	Le	MED-SKI: decoction for greasy hair	0	0	2	0	0	19	W	+
			MED-MUS: infusion or decoction against rheumatisms	3	0	0	1	0			
			MED-GEN: decoction against prostatitis and as diuretic	3	0	2	0	0			
			MED-MET: decoction or eaten raw as salad as blood depurative and against anemia	3	0	2	3	0			

*Legends*: *OTH*= others; *MI*= Mikrasiates; *PO*= Pontians; *DO*= Dopioi; *VL*=Vlachs. *CS*=cultivated species; *WS*= wild species; *Ap*=aerial part; *Bu*= bulb; *Ga*=galbula; *Fl*=flowers; *Fr*=fruits; *FrPed*= fruit pedicels; *Inf*= inflorescences; *Le*= leaves; *LeSh*=leaf shoots; *Re*=resin; *Ro*= roots; *Se*=seeds; *StFl*=styles of female flowers; *Tu*=tubers. *DOM*=domestic; *FOOD*=for human nutrition and health; *MED-CAR*=cardiovascular diseases; *MED-GAS*=gastrointestinal diseases; *MED-GEN*=genitourinary diseases; *MED-MET*=metabolic diseases; *MED-MUS*=muscular-skeletal diseases; *MED-NER*=nervous-sensorial diseases; *MED-RES*= respiratory diseases; *MED-SKI*=skin diseases. *∗* Reported in Hanlidou et al. [8] for at least one of the uses cited in the present study.

**Table 2:** Informant consensus factor.

Disease categories	Citations	Taxa	FIC
Respiratory diseases	154	24	0,85
Skin diseases	104	19	0,83
Cardiovascular diseases	39	9	0,79
Gastrointestinal diseases	75	18	0,77
Genitourinary diseases	65	17	0,75
Nervous-sensorial diseases	58	16	0,74
Metabolic diseases	59	18	0,71
Muscular-skeletal diseases	42	15	0,66
Total	596	136	

**Table 3:** Results of quantitative analysis for the most relevant species (≥ 8 citations). Disease categories with max FL are reported in bold.

	N informants citing	Total N citations	Cardiovascular diseases	Gastrointestinal diseases	Genitourinary diseases	Metabolic diseases	Muscular-skeletal diseases	Nervous-sensorial diseases	Respiratory diseases	Skin diseases	Max FL
*Hypericum perforatum *L.	47	66		15			4	2	1	**44**	67
*Matricaria chamomilla *L.	29	34		11	4			**14**	2	3	41
*Sideritis scardica *Griseb.	30	30							**30**		100
*Tilia platyphyllos *Scop.	23	27		4				1	**22**		81
*Sambucus nigra *L.	24	24							**20**	4	83
*Rosa canina *L.	16	20			1	**17**	2				85
*Urtica dioica *L.	11	19			5	**8**	4			2	42
*Mentha spicata *L.	13	18	5	**8**		5					44
*Achillea millefolium *L.	12	17			**10**				5	2	59
*Momordica charantia *L.	16	17		1						**16**	94
*Malva silvestris *L.	14	16		1		1			**14**		88
*Crataegus monogyna *Jacq.	12	13	**13**								100
*Dactylorhiza sp.*	13	13							**13**		100
*Linum usitatissimum *L.	9	13		3					**9**	1	69
*Paliurus spina-christi *Mill.	11	11			**10**	1					91
*Equisetum arvense *L.	7	10			**5**		**5**				50
*Plantago media *L.	9	10			2					**8**	80
*Verbascum longifolium *Ten.	8	10		2	2				**6**		60
*Arctium lappa *L.	5	8					**5**			3	63
*Micromeria juliana *(L.) Rchb.	7	8			**8**						100
*Origanum majorana *L.	8	8		**7**	1						88
*Satureja montana *L.* subsp. macedonica *(Formanek) Baden	8	8				1		3	**4**		50

## Data Availability

The ethnobotanical data used to support the findings of this study have been deposited in the Department of Pharmacognosy and Natural Products Chemistry, Faculty of Pharmacy, National and Kapodistrian University of Athens, Athens.
